# Transcription of Biotic Stress Associated Genes in White Clover (*Trifolium repens L*.) Differs in Response to Cyst and Root-Knot Nematode Infection

**DOI:** 10.1371/journal.pone.0137981

**Published:** 2015-09-22

**Authors:** Afsana Islam, Chris F. Mercer, Susanna Leung, Paul P. Dijkwel, Michael T. McManus

**Affiliations:** 1 Institute of Fundamental Sciences, Massey University, Palmerston North, New Zealand; 2 AgResearch Grasslands, Palmerston North, New Zealand; James Hutton Institute, UNITED KINGDOM

## Abstract

The transcription of four members of the *Kunitz proteinase inhibitor (KPI)* gene family of white clover (*Trifolium repens* L.), designated as *Tr-KPI1*, *Tr-KPI2*, *Tr-KPI4* and *Tr-KPI5*, was investigated at both local infection (roots) and systemic (leaf tissue) sites in white clover in response to infection with the clover root knot nematode (CRKN) *Meloidogyne trifoliophila* and the clover cyst nematode (CCN) *Heterodera trifolii*. Invasion by the CRKN resulted in a significant decrease in transcript abundance of *Tr-KPI4* locally at both 4 days post-infection (dpi) and at 8 dpi, and an increase in transcription of *Tr-KPI1* systemically at 8 dpi. In contrast, an increase in transcript abundance of all four *Tr-KPI* genes locally at 4 and 8 dpi, and an increase of *Tr-KPI1*, *Tr-KPI2*, and *Tr-KPI*5 at 8 dpi systemically was observed in response to infection with the CCN. Challenge of a resistant (R) genotype and a susceptible (S) genotype of white clover with the CCN revealed a significant increase in transcript abundance of all four *Tr-KPI* genes locally in the R genotype, while an increase in abundance of only *Tr-KPI1*, *Tr-KPI2*, and *Tr-KPI*5 was observed in the S genotype, and only at 4 dpi. The transcript abundance of a member of the*1-AMINOCYCLOPROPANE-1-CARBOXYLATE (ACC) SYNTHASE* gene family from white clover *(Tr-ACS1)* was significantly down-regulated locally in response to CRKN infection at 4 and 8 dpi and at 4 dpi, systemically, while abundance increased locally and systemically at 8 dpi in response to CCN challenge. Conversely, the abundance of the jasmonic acid (JA) signalling gene, *CORONATINE-INSENSITIVE PROTEIN 1* from white clover (*Tr-COI1*) increased significantly at 8 dpi locally in response to CRKN infection, but decreased at 8 dpi in response to CCN infection. The significance of this differential regulation of transcription is discussed with respect to differences in infection strategy of the two nematode species.

## Introduction

The mechanisms of plant defence against herbivory and microbial pathogens can be broadly classified as constitutive or induced. The induced defences include the biosynthesis of an array of secondary metabolites and proteins that can act as toxins, anti-feedents or anti-nutrients [1]. Of these, one of the most common inducible defences in plants against herbivory is the synthesis of proteinase inhibitors (PIs). The PI proteins are mostly low molecular mass proteins that occur in all life forms and these proteins are widely distributed through the kingdoms [2]. In all organisms, PIs are classified into super-families based on the class of proteinase inhibited to give serine, aspartic, metallo- and cysteine-proteinase inhibitors [3]. One group of the serine PI superfamily are the Kunitz proteinase inhibitors (KPIs) which, where examined in plants, are widespread and generally exist as multi-gene families [4–7]. In common with the large PI families in plants, the KPIs have been shown to function as storage proteins and insect pest resistance factors [8–11], and have been used in transgenic approaches to confer protection against insect pests to the transformants [12–16].

In terms of the other herbivorous pests of plants, parasitic nematodes also cause significant crop losses globally [17], with over 4100 species identified thus far [18]. Of relevance to this study are two groups; the root-knot nematodes (RKN), of the genus *Meloidogyne* from the family Meloidogynidae, and the cyst nematodes (CN) comprising two genera, *Heterodera* and *Globodera*, from the family Heteroderidae. In terms of infection strategy, the two groups share some similarities. An infective and motile juvenile second stage (J2) invades host roots near the tips and establishes a feeding site in the vasculature/inner cortex region before becoming sedentary. However, significant differences also exist between the two groups in terms of migration pathways through the roots and the mechanism of feeding site formation. RKN J2 migrate intercellularly in host roots, while CN J2 move intracellularly. The RKN feeding sites are composed of giant cells while CN feeding sites are syncytia, formed by the fusions of hundreds of syncytial initials. During the infection and parasitism process, extensive changes in the expression of host genes are observed [19–25] which are, in part, in response to nematode invasion and to stylet secretion proteins, termed effectors [26–28].

The major secretory proteins from nematodes include the cysteine proteinases, and so the accumulation of cysteine PIs (cystatins) in transgenic backgrounds has been reported to confer a degree of resistance against CN and RKN in tomato [29,30], *Arabidopsis* [31,32], rice [33], potato [34,35], alfalfa [36], banana [37] and sweet potato [38]. However, in contrast to the cystatins, there are fewer instances where serine PIs have been shown to exert the same effects in transgenic backgrounds against nematode pests [31,39]. Much less is known too in terms of the interactions of nematode feeding and the KPIs. In transgenic studies, over-expression of the sporamin gene from sweet potato in sugar beet did reduce the growth and development of the sugar beet cyst nematode, *H*. *schachtii* [40]. More directly, infection by the soybean cyst nematode, *H*. *glycines*, was shown to differentially regulate the expression of a family of *KPI* genes which was dependent on the genetic background of soybean [41], while the infection of potato with the potato cyst nematode, *G*. *rostochiensis*, also influenced the expression of a small *KPI* gene family [42].

In the pasture legume white clover (*Trifolium repens* L.), four full-length *KPI* genes, designated *Tr‐KPI1*, *Tr‐KPI2*, *Tr‐KPI4* and *Tr‐KPI5*, have been isolated, and these have been shown to display differential transcription during seed germination, in different tissues of the mature plant, and transcription has also been shown to be ontogenetically-regulated [43]. Further, both wounding and insect feeding of leaf tissue by the generalist insect herbivore, *Spodoptera litura*, differentially regulates the transcription of the *KPI* gene family both locally at the site of insect damage and in remote tissue, supporting a systemic response [43]. Over-expression of *Tr-KPI1*, *Tr-KPI2*, *Tr-KPI4* and *Tr-KPI5* in *Nicotiana tabacum* retarded larval growth of feeding *S*. *litura* [43]. However, the influence of nematode infection has not been evaluated in terms of any such differential expression.

In New Zealand, species of root-knot nematode (RKN) and a species of cyst nematode (CN), *H*. *trifolii* are parasites of white clover in pastures [44]. Thus here two different root parasites, the clover CN (CCN), *H*. *trifolii* and the clover RKN (CRKN) *M*. *trifoliophila*, have been used for a comparative study on changes in transcript abundance of the *Tr-KPI* gene family using a single white clover genotype of the cv. Huia that is susceptible to both nematodes. In addition, both CN-susceptible and CN-resistant genotypes obtained from a white clover breeding programme were used to examine any genotype-dependent regulation. To expand our study on the significance of the observed transcriptional changes that occur within the *Tr-KPI* gene family in response to nematode infection, we also examined the transcript abundance of two key genes associated with biotic stress responses, including plant-nematode interactions. The first was *1-AMINOCYCLOPROPANE-1-CARBOXYLATE (ACC) SYNTHASE* from white clover *(Tr-ACS1)*, coding for the enzyme that is recognised as the rate-determining step in ethylene (ET) biosynthesis [45], while the second was *CORONATINE-INSENSITIVE PROTEIN 1* from white clover (*Tr-COI1*) that encodes a component of the JA signalling pathway [46]. In total, changes in the transcript abundance of all genes examined were evaluated in terms of the documented differences in infection strategy of the two nematode species.

## Materials and Methods

### Plant material

A single genotype (i.e. arising from a single seed) of the white clover cultivar Grassland ‘Huia’ (AgResearch Grasslands, Palmerston North, New Zealand) that was susceptible to both CRKN and CCN infection (designated as a double susceptible white clover genotype of cv. Huia) was used in the infection experiments with both CRKN and CCN. In separate experiments, genotypes resistant (17R) or susceptible (23S) to the CCN were used; these genotypes were selected from two breeding lines originating four generations earlier from the same line. These lines were from an AgResearch recurrent selection programme developing resistance to CCN in white clover by selecting germplasm supporting fewer cysts, where the numbers of eggs per cyst were concurrently reduced [47].

To maintain each genotype, plant material was propagated vegetatively through stolon cuttings; the apical part of the stolon was excised just proximal to node 4 and all leaves, except the first emerged leaf, were removed. The cuttings were then placed, by burying the basal two nodes, into pots containing either vermiculite or peat-based potting mix followed by regular watering with half-strength Hoagland’s solution [48] until rooting had occurred. These cuttings were then maintained in a temperature-controlled glasshouse (minimum 20°C; venting at 25°C) until sufficient growth had occurred to generate well-established stock plants.

### Root infection experiments

The nematode infection experiments were performed using stolon cuttings (obtained as described) excised from a double-susceptible white clover genotype of cv. Huia or the resistant (17R) and susceptible (23S) genotypes, as appropriate. The stolon cuttings from the stock plants were rooted in peat-based potting mix in 300 x 450 mm trays and maintained in a temperature-controlled glasshouse (minimum 20°C; venting at 25°C) on a plant-heating mat for 20 days. Well-established stolons were then selected and transplanted into 60-mm-diameter, 156-mL capacity plastic cups containing pasteurised sand and were watered with half-strength Hoagland’s solution on a regular basis. To infect the plants, a hole was made in the middle of each cup and inoculum was added at a rate of 4000 eggs/3 mL of water for *M*. *trifoliophila* and 3000 eggs/3 mL of water for *H*. *trifolii*. An identically propagated set of plants that were not infected with nematodes served as the control group. Both whole roots (designated the local tissue) and the first fully expanded (FFE) leaf on the stolon (designated the systemic tissue) were harvested from both infected and control plants after 4 and 8 dpi, immediately frozen in liquid nitrogen and stored at -80°C until RNA isolation. To check infection of the plants, four extra plants of the double-susceptible genotype were infected with both CCN and CRKN and the roots were stained with aniline blue at 4 dpi. To do this, infected roots were washed in running tap water, placed in 1.5% (v/v) NaOCl for 5 min with agitation, rinsed again in running tap water for 30 sec and allowed to stand in tap water for 15 min and blot dried. The roots were then incubated in a boiling solution [0.05% (w/v) aniline blue in 33% (v/v) glycerol, 33% (v/v) lactic acid] for 1 min, cooled to room temperature, briefly rinsed in running tap water and then blot dried. The stained nematodes were visualized using a Zeiss Axiophot photomicroscope.

### Nucleic acid isolation and cDNA synthesis

Total RNA was extracted using the Hot Borate method of [49] and [50]. The RNA concentration was determined using a NanoDrop ND-1000 spectrophotometer V3.6 (Thermo Scientific, USA). Genomic DNA-free RNA samples were prepared using an RNase-free recombinant DNase treatment (Roche). To synthesize the first single strand DNA, the Transcriptor First Strand cDNA synthesis kit (Roche) was used using Oligo (dT)_18_ primers. For this, total RNA (1 μg) was combined with Oligo (dT)_18_ primer in 0.2 mL capacity tubes and the volume was adjusted to 13 μL with water. The RNA and primers were denatured at 65°C for 10 min and placed on ice immediately. Seven μL of master reaction mixture containing 5X transcriptor RT reaction buffer, protector RNase inhibitor (40 U/μL), 10 mM dNTP-Mix and Transcriptor reverse transcriptase (20 U/μL) was then added. The tubes were placed in the thermocycler and, typically, cDNA synthesis was carried out at 55°C for 30 min, before inactivation of the reverses transcriptase at 85°C for 5 min.

### Quantitative reverse-transcription PCR (qRT-PCR)

For qRT-PCR analysis, specific primers (S1 Table) were designed according to the general requirements of qRT-PCR primers [T_m_ = 60°C (± 1°C), a minimal secondary structure, and an inability to form stable dimers] and based on the cDNA sequences for the representative target genes (S2 Table). The efficiency of all the primers was determined by the standard curve method [51]. qRT-PCR was performed using the LightCycler^®^ 480 Real-Time PCR (Roche) and system series software 1.7, with three technical replicates of each cDNA sample (20-fold dilution). SYBR green I was used to monitor efficient DNA synthesis. Typically a 10 μL of reaction volume was used that consisted of 5 μL of 2 X LightCycler^®^ 480 SYBR Green I Master Mix (Roche), 2.5 μL of 20-fold diluted cDNA and 0.5 μL of 10 μM forward and reverse primers. Master mixture and cDNA templates were dispensed into 96-well plates. The PCR was performed as follows; 95°C for 5 min (95°C 10 sec, 60°C 10 sec, 72°C 10 sec) x 40 cycles, 95°C melt. Fluorescence measurements were performed at 72°C for each cycle and continuously during final melting. Relative transcript abundance was determined by comparative quantification to the geometric mean using three biological replicates, where one individual plant represents a single biological replicate. Two or three independent qRT-PCR reactions were performed per replicate and transcript abundance was normalized using two internal reference genes, *Tr-β-ACTIN* and *Tr-GAPDH*. The reference genes were selected using BestKeeper [52] and the efficiency of all primers was determined by the LinRegPCR quantitative PCR data analysis program [51]

### Statistical analysis

Statistical analysis was performed using the Statistical Package for the Social Sciences (IBM SPSS Statistics) and any significant differences determined using a pairwise Student’s t-test for 4 and 8 dpi separately as the transcript abundance of *Tr-KPIs* are developmentally regulated in white clover [43]. The Holm-Bonferroni method was also used for the application of a multiple testing comparison for the assessment of multiple gene analysis from the same cDNA sample [53].

## Results

### Infection time-course of root knot and cyst nematodes in white clover roots

**Fig 1:**
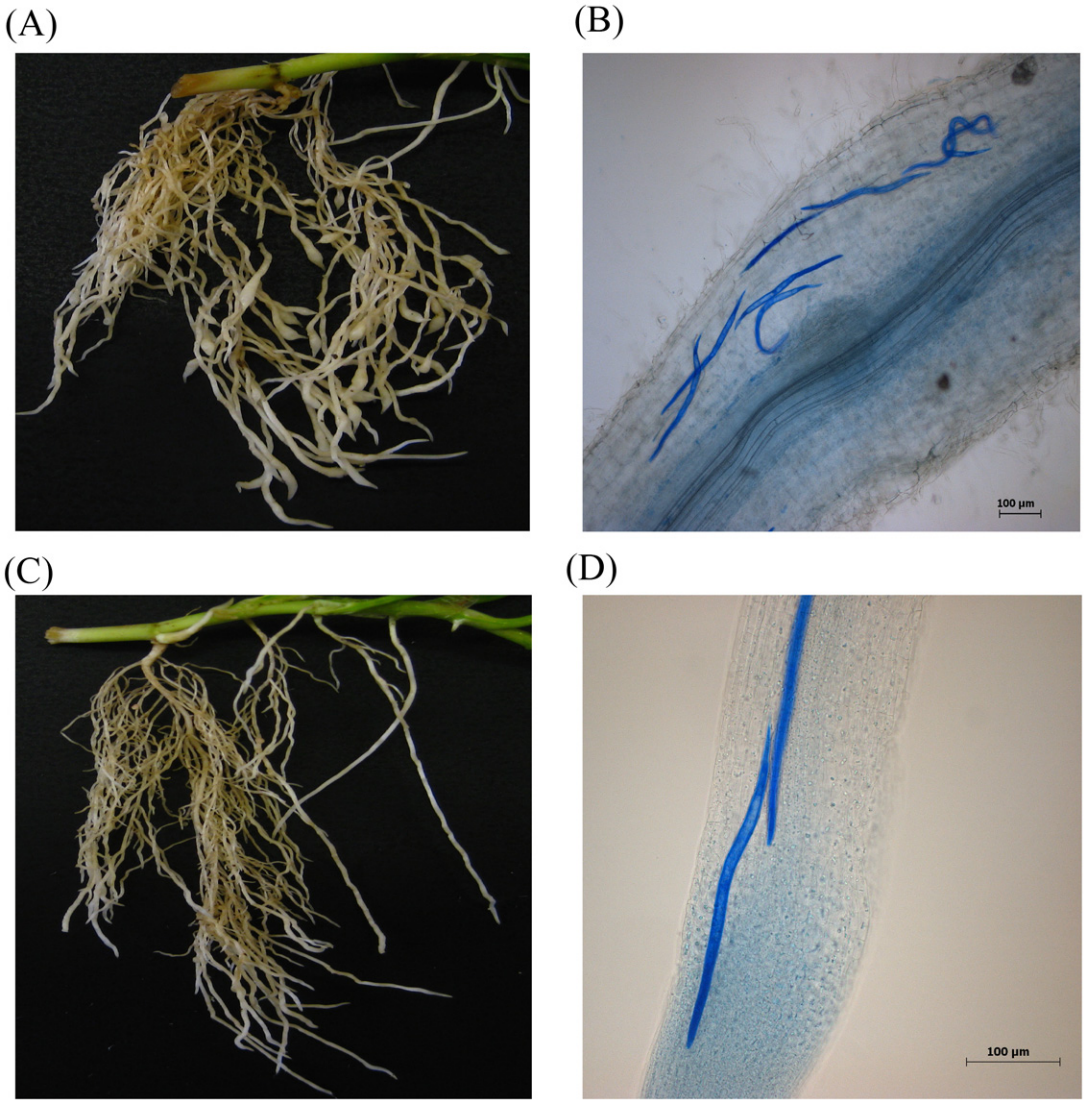
Representative images of the root system of a double-susceptible white clover genotype of cv. Huia at 4 dpi after infection with CRKN *Meloidogyne trifoliophila* (A, B) and CCN *Heterodera trifolii* (C, D). Nematodes were visualized inside root tissue with 0.05% (v/v) aniline blue staining (B, D). Images in (A) and (C) are captured at 1 X magnification. Images in (B) and (D) are captured at 50 x magnification; scale bar = 100 μm.

By 4 dpi, the eggs of both nematode species were hatched and invasion of root tissue of the double-susceptible genotype of cv. Huia was evident (Fig 1). Infection with CRKN eggs had produced discernible swelling at the root tips. In both infections, aniline blue-stained J2 were observed near the inner cortex of the root at 4 dpi suggesting successful egg hatching and J2 invasion. By 8 dpi, gall formation and giant cell formation was visible suggesting active feeding (data not shown).

### CRKN and CCN infections influence the transcript abundance of the *Tr-KPI* gene family

**Fig 2:**
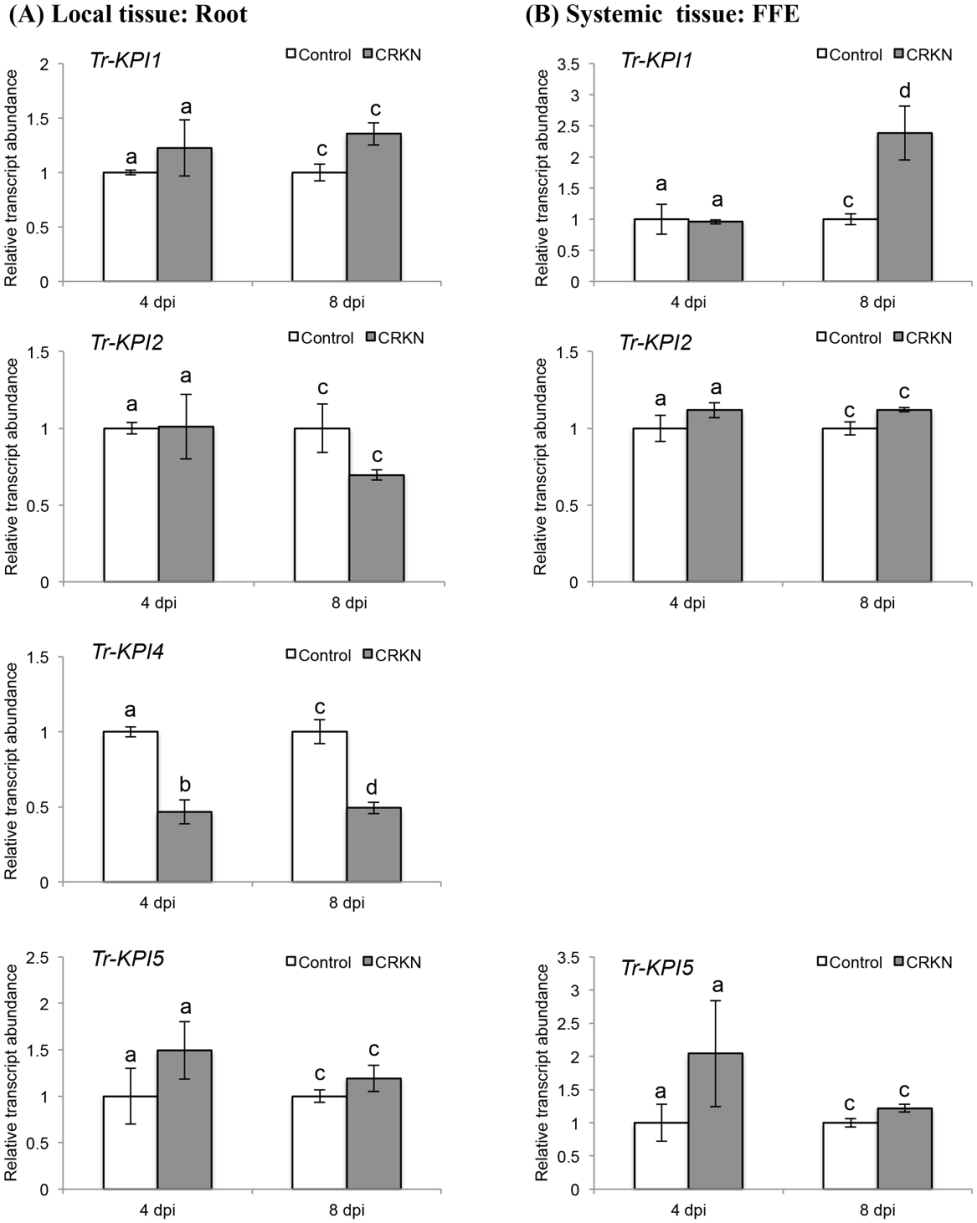
CRKN infection of a double-susceptible white clover genotype of cv. Huia influences the transcript abundance of the *Tr-KPI* gene family. (A) Changes in *Tr-KPI* transcript abundance in root tissue excised from either control or CRKN-infected plants, as indicated, and at the times indicated. (B) Changes in *Tr-KPI* transcript abundance in the first fully expanded (FFE) leaf tissue excised from either control plants or plants with CRKN-infected roots, as indicated, and harvested at the times indicated. Bars represent the mean values, ± SEM, of three biological replicates (n = 3). The control for each data point was set to 1. The letters a and b indicate statistically significant differential expression at 4 dpi and the letters c and d indicate statistically significant differential expression at 8 dpi when compared with the respective control plants that were not challenged with CRKN eggs; the same letter above the control and treatment bars indicate insignificant difference.

**Fig 3:**
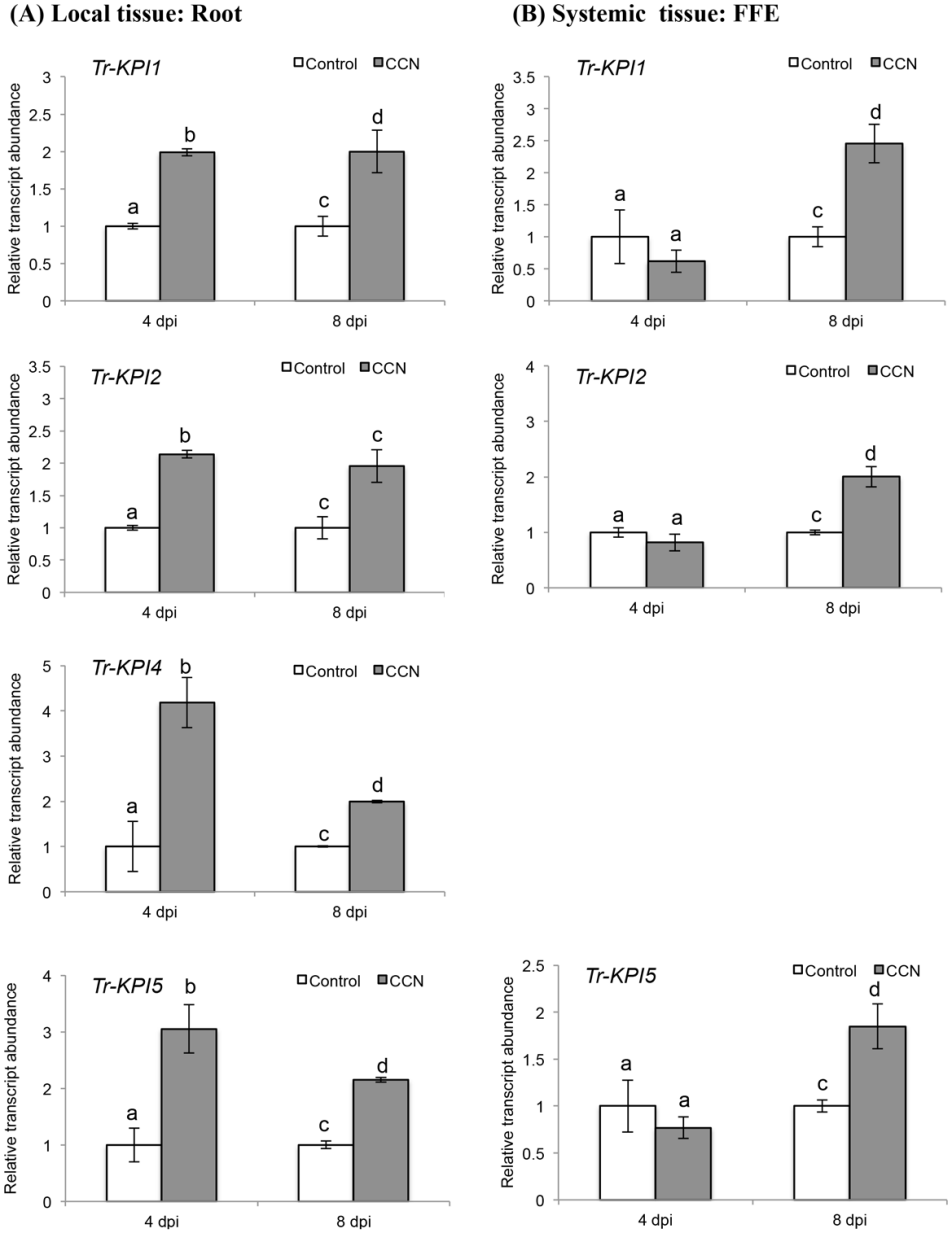
CCN infection of a double-susceptible white clover genotype of cv. Huia influences the transcript abundance of the *Tr-KPI* gene family. (A) Changes in *Tr-KPI* transcript abundance in root tissue excised from either control or CCN-infected plants, as indicated, and at the times indicated. (B) Changes in *Tr-KPI* transcript abundance in the first fully expanded (FFE) leaf tissue excised from either control plants or plants with CCN-infected roots, as indicated, and harvested at the times indicated. Bars represent mean values, ± SEM, of three biological replicates (n = 3). The control for each data point was set to 1. The letters a and b indicate statistically significant differential expression at 4 dpi and the letters c and d indicate statistically significant differential expression at 8 dpi when compared with the respective control plants that were not challenged with CCN eggs; the same letter above the control and treatment bars indicate insignificant difference.

The transcript levels of the four distinct *Tr‐KPI* genes in response to CRKN and CCN infection of the double-susceptible genotype of cv. Huia were investigated initially. In response to CRKN, the transcript abundance of all four *Tr-KPI* genes in the infected root tissue (locally) did not show any significant increase when compared with the control (uninfected) tissue (Fig 2A), but a significant increase (*p* = 0.010) in the abundance of *Tr-KPI1* only was observed at 8 dpi in the remote FFE leaf tissue (Fig 2B). However, a significant decrease in the abundance of the root-specific *Tr-KPI4* gene was observed locally at 4 dpi (*p* = 0.010) and 8 dpi (*p* = 0.038) (Fig 2A). In contrast to the CRKN response, a significant increase in transcript abundance of all four *Tr-KPI* genes was observed locally at both 4 dpi (*Tr-KPI1*, *p* = 0.015; *Tr-KPI2*, *p* = 0.0001; *Tr-KPI4*, *p* = 0.010; *Tr-KPI5*, *p* = 0.0177) and 8 dpi (*Tr-KPI1*, *p* = 0.012; *Tr-KPI2*, *p* = 0.037; *Tr-KPI4*, *p* = 0.021; *Tr-KPI5*, *p* = 0.019) in response to CCN infection (Fig 3A). The increase in the transcript abundance of *Tr-KPI2* in response to CCN infection was also significant using the Holm-Bonferroni multiple testing correction (Fig 3A). However, for *Tr-KPI4*, a significant decrease in transcript abundance was observed at 8 dpi when compared with 4 dpi (Fig 3A). In the remote FFE leaf tissue, a significant increase in abundance was also observed for *Tr-KPI1* (*p* = 0.004), *Tr-KPI2* (*p* = 0.018) and *Tr-KPI5* (*p* = 0.023), but only at 8 dpi (Fig 3B).

### The transcription of the *Tr-KPI* gene family is differentially regulated in resistant and susceptible lines in response to CCN infection

**Fig 4:**
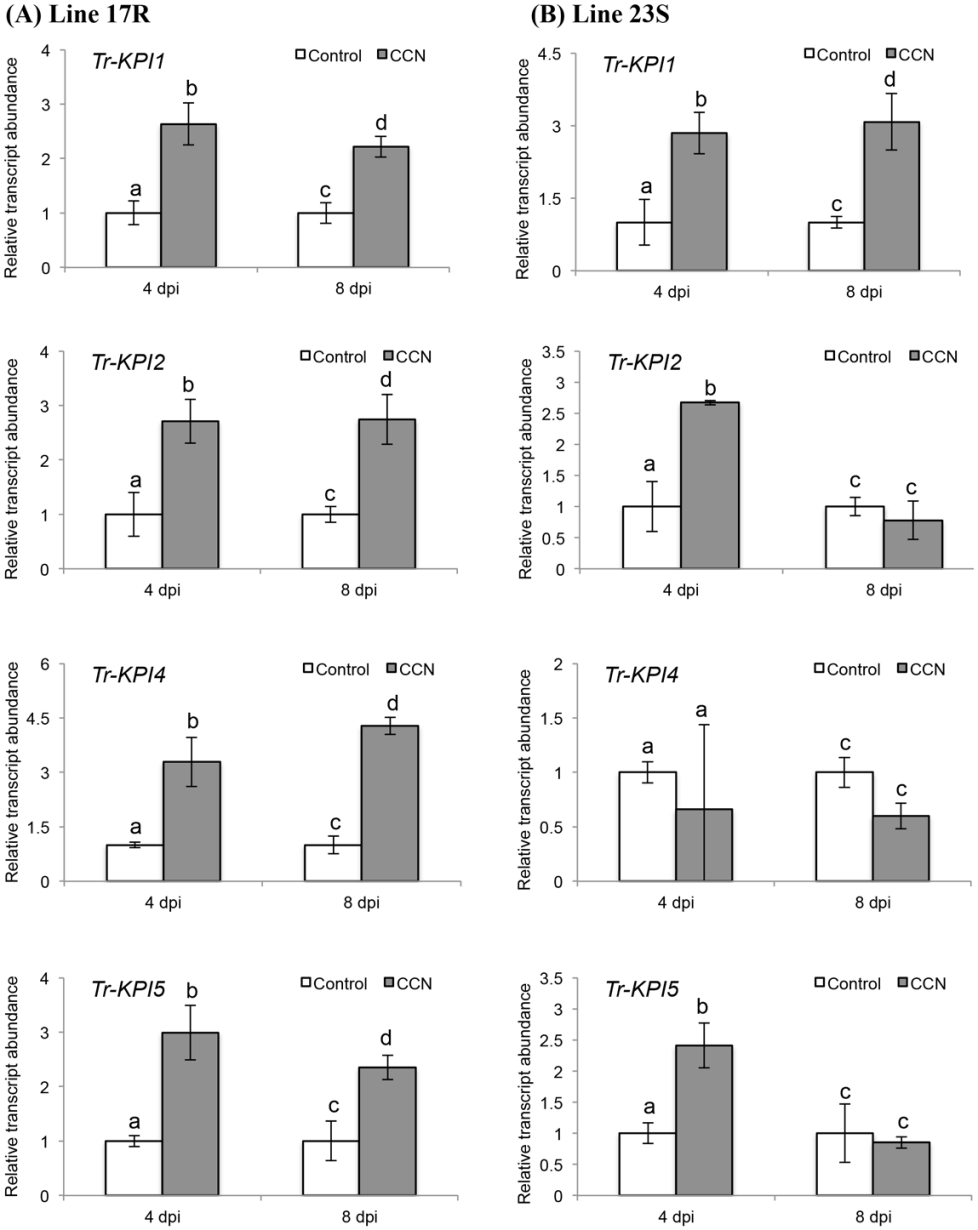
CCN infection influences the transcript abundance of the *Tr-KPI* gene family in a CCN-resistant genotype (17R) and a CCN-unsusceptible genotype (23S) of white clover cv. Huia. Changes in *Tr-KPI* transcript abundance in root tissue excised from either control or CCN-infected 17R (A) or 23S (B) plants, as indicated, and at the times indicated. Bars represent mean values, ± SEM, of three biological replicates (n = 3). The control for each data point was set to 1. The letters a and b indicate statistically significant differential expression at 4 dpi and the letters c and d indicate statistically significant differential expression at 8 dpi when compared with the respective control plants that were not challenged with CCN eggs; the same letter above the control and treatment bars indicate insignificant difference.

A marked increase in transcript abundance of all members of the *KPI* gene family was observed in the double-susceptible genotype of cv. Huia in response to CCN infection (Fig 3). To examine this in more detail, two genotypes of white clover from a nematode resistance breeding programme were assessed. Data for cyst numbers on four genotypes derived from the breeding programme is given in S3 Table, from which a resistant (17R) and a susceptible (23S) genotype were then selected for comparison. In response to CCN infection, a significant increase in the transcript abundance of all four *Tr-KPI* genes was again observed locally in the 17R line at both 4 dpi (*Tr-KPI1*, *p* = 0.013; *Tr-KPI2*, *p* = 0.023; *Tr-KPI4*, *p* = 0.013; *Tr-KPI5*, *p* = 0.015) and 8 dpi (*Tr-KPI1*, *p* = 0.025; *Tr-KPI2*, *p* = 0.021; *Tr-KPI4*, *p* = 0.0011; *Tr-KPI5*, *p* = 0.042) (Fig 4A). For the 23S line, a significant increase in the transcript abundance of *Tr-KPI1* at both 4 dpi (*p* = 0.031) and 8 dpi (*p* = 0.011) was observed, but for *Tr-KPI2* and *Tr-KPI5* this significant increase was only observed at 4 dpi (*Tr-KPI2*, *p* = 0.011; *Tr-KPI5*, *p* = 0.021). No significant differences for *Tr-KPI4* could be discerned at either time-point evaluated (Fig 4B).

### Effect of nematode infection on the transcript abundance of ET biosynthetic and JA signalling genes

**Fig 5:**
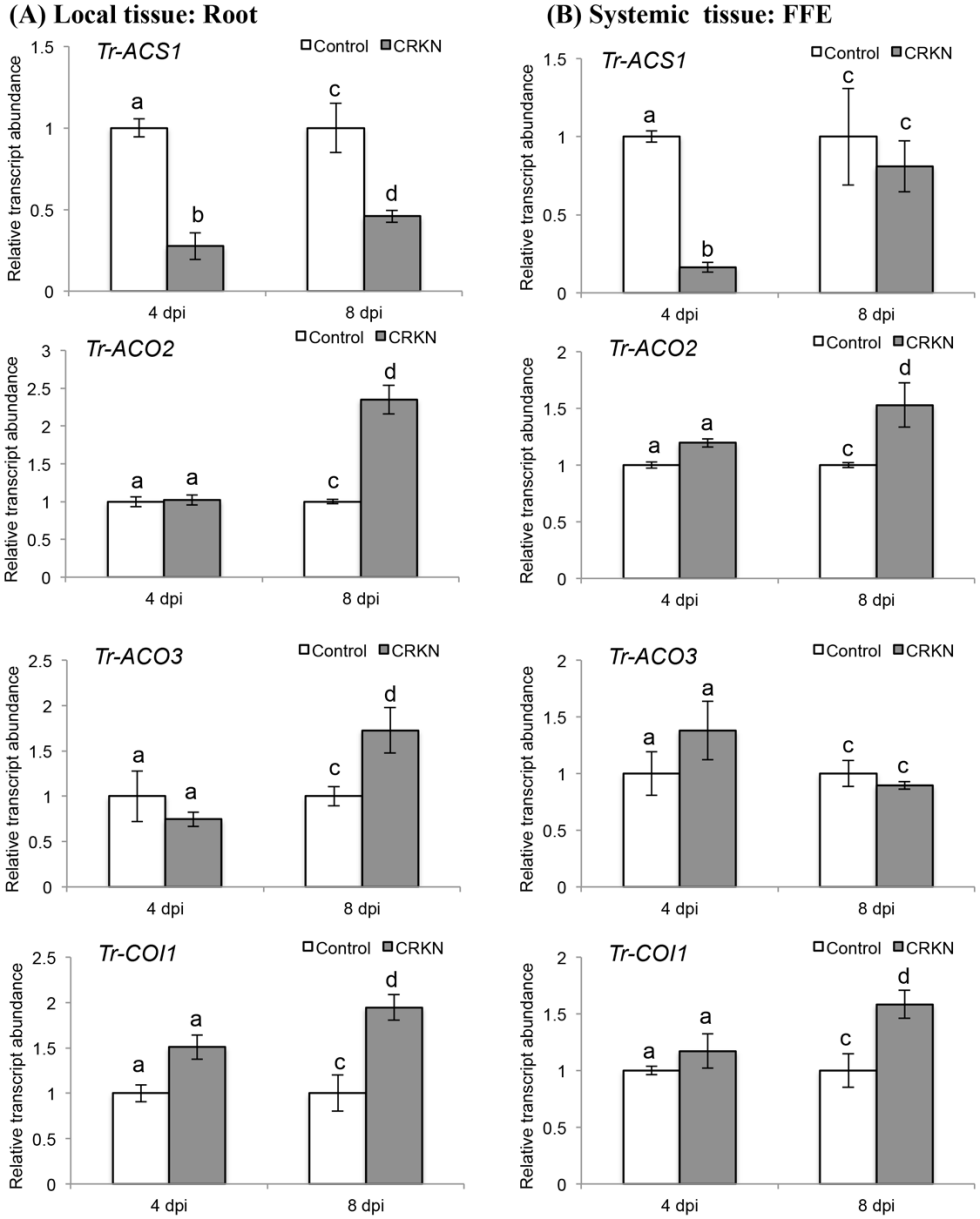
CRKN infection of a double-susceptible white clover genotype of cv. Huia influences the transcript abundance of ET biosynthetic and JA signalling genes. (A) Changes in transcript abundance of *Tr-ACS1*, *Tr-ACO2*, *Tr-ACO3* and *Tr-COI1*, as indicated, in roots excised from control or CRKN-infected plants, as indicated, at the times indicated. (B) Changes in transcript abundance of *Tr-ACS1*, *Tr-ACO2*, *Tr-ACO3* and *Tr-COI1*, as indicated, in the first fully expanded (FFE) leaf tissue excised from control plants or plants with CRKN-infected roots, as indicated, at the times indicated. Bars represent mean values, ± SEM, of three biological replicates (n = 3). The control for each data point was set to 1. The letters a and b indicate statistically significant differential expression at 4 dpi and the letters c and d indicate statistically significant differential expression at 8 dpi when compared with the respective control plants that were not challenged with CRKN eggs; the same letter above the control and treatment bars indicate insignificant difference.

For the ET biosynthetic genes examined in response to CRKN infection of the double-susceptible genotype, a significant decrease in the transcript abundance of *Tr-ACS1* was observed locally (in the root tissue) at 4 dpi (*p* = 0.0005) and 8 dpi (*p* = 0.012), and at 4 dpi (*p* = 0.001) systemically (in the FFE tissue) (Fig 5A and 5B). The decrease in *Tr-ACS1* transcript abundance at 4 dpi in both the local and systemic tissues was also shown to be significant using the Holm-Bonferroni correction. A significant increase in the transcript abundance of white clover *ACC OXIDASE* (*ACO*) genes, *Tr-ACO2* (*p* = 0.005) and *Tr-ACO3* (*p* = 0.037), was observed locally at 8 dpi (Fig 5A), and for *Tr-ACO2* systemically at 8 dpi (*p* = 0.028; Fig 5B) with the increase in transcript abundance of *Tr-ACO2* locally at 8 dpi also shown to be significant using the Holm-Bonferroni correction (Fig 5A). In contrast to the decrease in transcript abundance of *Tr-ACS1*, a significant increase in the abundance of *Tr-COI1* was observed both locally (*p* = 0.019; Fig 5A) and systemically at 8 dpi (*p* = 0.040; Fig 5B).

**Fig 6:**
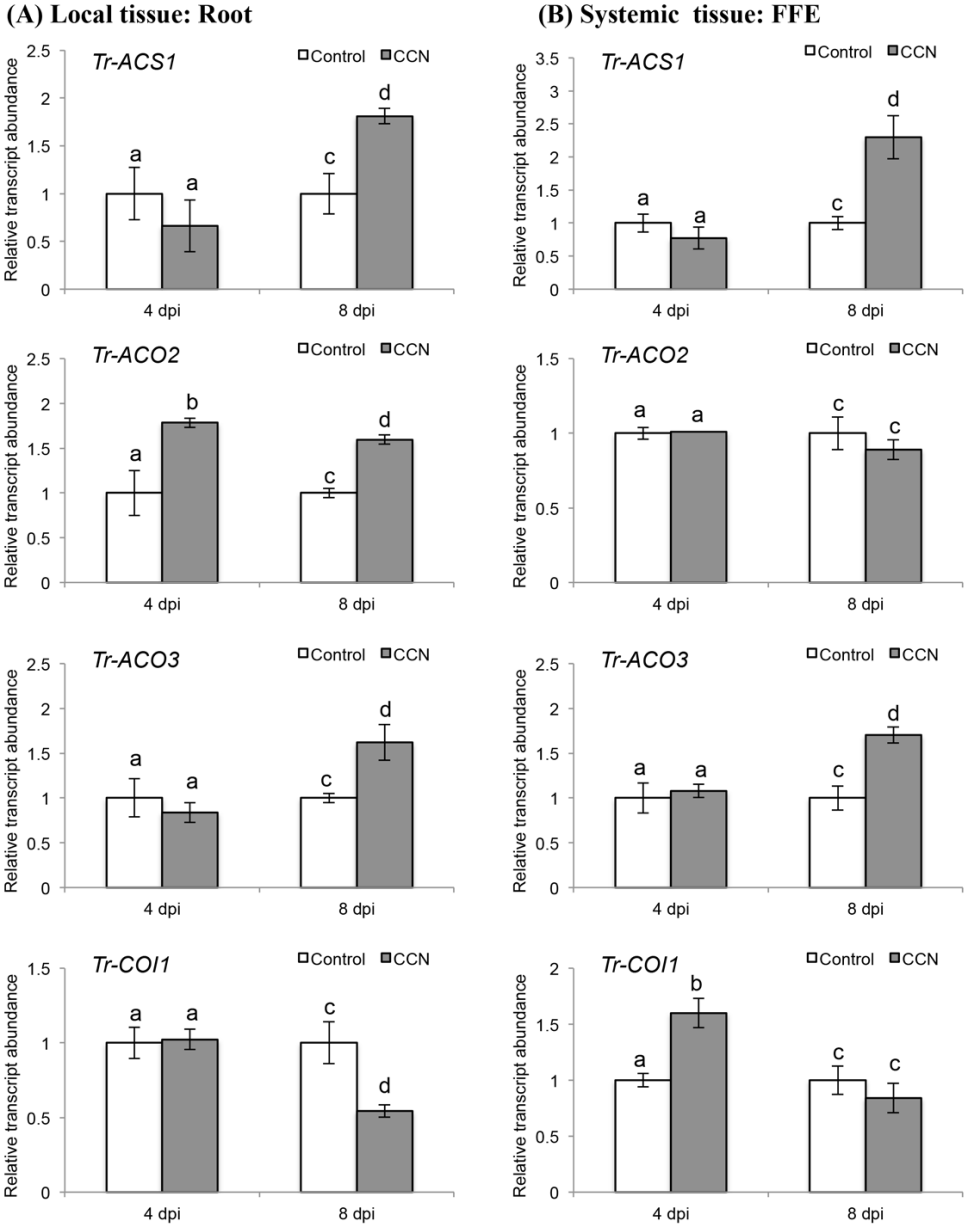
CCN infection of the double-susceptible white clover genotype of cv. Huia influences the transcript abundance of ET biosynthetic and JA signalling genes. (A) Changes in transcript abundance of *Tr-ACS1*, *Tr-ACO2*, *Tr-ACO3* and *Tr-COI1*, as indicated, in roots excised from control or CCN-infected plants, as indicated, at the times indicated. (B) Changes in transcript abundance of *Tr-ACS1*, *Tr-ACO2*, *Tr-ACO3* and *Tr-COI1*, as indicated, in the first fully expanded (FFE) leaf tissue excised from control plants or plants with CCN-infected roots, as indicated, at the times indicated. Bars represent mean values, ± SEM, of three biological replicates (n = 3). The control for each data point was set to 1. The letters a and b indicate statistically significant differential expression at 4 dpi and the letters c and d indicate statistically significant differential expression at 8 dpi when compared with the respective control plants that were not challenged with CCN eggs; the same letter above the control and treatment bars indicate insignificant difference.

For CCN infection, a significant increase in *Tr-ACS1* transcript abundance was observed both locally (*p* = 0.040) and systemically (*p* = 0.018) at 8 dpi (Fig 6A and 6B), while a significant increase in the abundance of *Tr-ACO2* was observed at both 4 dpi (*p* = 0.028) and 8 dpi (*p* = 0.014), but only locally (Fig 6A). For *Tr-ACO3*, a significant increase in transcript abundance was observed both in local (*p* = 0.025) and systemic (*p* = 0.012) tissue at 8 dpi (Fig 6A and 6B). For *Tr-COI1*, a significant decrease in transcript abundance was observed locally at 8 dpi (*p* = 0.032; Fig 6A), but abundance increased significantly at 4 dpi (*p* = 0.013) in the remote FFE leaf tissue (Fig 6B).

## Discussion

This study has sought to examine the effect of nematode infection strategies on the transcript abundance of the *KPI* gene family from white clover, as well as any changes in the transcription of key biotic-stress-associated hormone biosynthetic and signalling genes.

The interactions of PIs and nematodes has been well studied, largely based on the utilisation of these factors for resistance in transgenic plants. While some serine PIs, including Kunitz proteins, have been tested in this way [31,39,40], the larger focus has been on the cystatins as this gene family is more commonly down-regulated during parasitism [19,21,22,24]. For the *KPI* genes, more targeted transcriptional studies have identified changes where the magnitude of the response can be dependent upon a resistant or susceptible background [41], but no comparative study of CN and RKN infection of the same species has been undertaken. It is well established that the *KPI* gene family is differentially regulated by wounding [54, 55] in plants including white clover [43] and so a comparative examination of changes in the transcript abundance of the *Tr-KPI* gene family can contribute to the elucidation of the differences in the infection strategies between the two groups of nematodes.

RKN parasitic J2 are known to migrate apoplastically through the root cortex towards the vascular tissue where typically 5–7 parenchyma cells adjacent to the xylem elements are selected as founder cells for the development of the giant cells. The giant cells are formed by synchronous nuclear divisions without cytokinesis to form large, multinucleate cells, after which a hyperplasia of pericycle and cortex cells form the root knots/galls [56]. During the intercellular migration process, little damage to the roots occurs and so a signature of the RKN-host interaction is that many of the defence-associated genes in the host are not induced [19, 26]. For white clover, the transcript abundance of the *Tr-KPI* genes was not increased locally, particularly at 4 dpi. Indeed the transcription of the root-specific *Tr-KPI4* gene was repressed. This is broadly in agreement with de Sa *et al*. [25] who did not detect any changes in the transcript abundance of a *KPI* gene expressed in the *M*. *javanica*—soybean interaction, although transcription across the larger gene family was not examined.

For the CN interaction, a different infection strategy is invoked. Here, J2 migrate intracellularly, thus causing cellular damage, until they reach the inner cortex where initial syncytial cells (ISC) are established. Unlike the giant cells, the syncytium develops by the coalescence of surrounding ISC that have originated from cortical cells. Together, this infection strategy suggests that plant responses to cell wounding and cell fusion are invoked, and microarray and transcriptome data highlight an increase in the expression of cell wall dissolution enzymes and an induction of the proteasome components [20, 22]. The *KPI* expression data obtained in this study support a wound response to CCN infection with a marked increase in transcript abundance of all of the *Tr-KPI* genes locally at both 4 dpi and 8 dpi and *Tr-KPI1*, *Tr-KPI2* and *Tr-KPI5* systemically at 8 dpi. These changes are very much in contrast to the changes in the CRKN interaction, but agree with the CN-*KPI* interactions reported by Rashed *et al*. [41] and Turra *et al*. [42].

The white clover genotype used in the CRKN and CCN comparative infection study was confirmed to be susceptible to both species of nematode (data not shown). In the CRKN interaction, no marked changes in transcript abundance of the *TR-KPI* gene family were observed (with the exception of a local repression of *Tr-KPI4*) suggesting that the *Tr-KPI* gene family are part of the defensive array in white clover. In contrast, the up-regulation of the gene family in response to CCN infection may reflect a highly altered cellular homeostasis caused by wounding which, in common with other wound responses, is accompanied by the induction of *Tr-KPI* genes in the systemic tissue (albeit at 8 dpi). In another study, *KPI* gene expression was measured during a CN interaction in soybean root and a greater induction was noted in the roots of susceptible plants when compared with those of resistant plants, although this was over 0.5–1 dpi [41]. In the current study, a resistant and susceptible genotype of white clover were compared, and while a more marked induction of *Tr-KPI* gene expression was observed in the resistant genotype (albeit only at 8 dpi) a significant induction was also observed in the susceptible genotype. These data thus suggest that the *KPI* gene response may not be a primary determinant in the resistance interaction, but more likely reflects the degree of cellular wounding and the corresponding magnitude of the metabolic changes that occur within the infected plants.

Given the difference in cellular homeostasis induced by the CRKN and CCN infection, as presaged by the differences in *Tr-KPI* transcription, it was also of interest, therefore, to examine the transcription of the key ET biosynthetic and JA signalling genes, the two hormones intimately linked to the regulation of plant responses to both biotic and abiotic stresses, including plant CN and RKN interactions [57–59]. In the CRKN interaction, the gene encoding for the rate-determining step in ET biosynthesis, *Tr-ACS1* was significantly repressed both locally and systemically at 4 dpi, with the decrease also significant using multiple testing correction thus supporting a more convincing difference. While *Tr-ACS1* was also repressed at 8 dpi locally, it was not so systemically, and also there was a local induction of the two ET precursor *ACO* genes examined at this later time point. Indeed, for *Tr-ACO2*, this difference was also shown to be significant using multiple testing correction. While we did not measure ET production directly, these changes in transcript abundance suggest a biphasic control of the biosynthetic pathway, with reduction of biosynthesis initially (at 4 dpi) and then a later induction (at 8 dpi). In rice, a decrease in *OsACS1* expression has also been observed in response to infection with *M*. *graminicola* in both local and systemic tissues [59]. Further, the addition of ET to aerial plant plants, *via* the application of the synthetic ET precursor 2-chloroethylphosphonic acid (ethephon or ethrel), induced a systemic defence response in the roots, as determined by the induction of the pathogenesis-related genes, *PR1a* and *PR1b*. These treated plants showed a reduction in the infection of the RKN, *M*. *graminicola* [58]. Further, our data support other approaches where the use of L-α-[2-(2-aminoethyoxy)vinyl]glycine (AVG), an inhibitor of ACC synthase activity, increased the attractiveness of *Arabidopsis* to the RKN, *M*. *hapla* [60]. Likewise in the same study, the ET over-producing mutants, *eto1*, *eto2* and *eto3* were deemed to be less attractive to the nematode. In earlier studies with the interaction between *M*. *javanica* and tomato roots, an increase in ET production was observed, but at 5 dpi [61]. If the natural ET precursor, ACC or the synthetic precursor compound ethrel were added, the gall number increased while added AVG decreased gall number. These workers proposed that ET served to aid cell expansion and subsequent gall formation. From our results, we can speculate for white clover that the later increase in *ACO* (after 8 dpi) may catalyse the production of ET that is important in gall formation. In contrast, the earlier suppression of *Tr-ACS1* (at 4 dpi) may be important at decreasing ET production and so promote the initial penetration by CRKN.

In contrast to the RKN interaction in *Arabidopsis*, the addition of ACC increased the susceptibility to infection by the beet cyst nematode, *H*. *schachtii*, as determined by an increase in attractiveness of infective juveniles for the root exudates and the addition of the ACC synthase inhibitor, AVG, decreased susceptibility [62]. Likewise, studies with ET over-producing mutants of *Arabidopsis* also resulted in an increase in susceptibility, while ET insensitive mutants displayed a decrease in susceptibility [62]. In the interaction between *H*. *glycines* and soybean roots, an increase in ACC content was observed in the roots in response to infection, which was accompanied by the induction of a suite of ACS genes from the multi-gene family [63]. For white clover, the observed local increase in *Tr-ACS* and *Tr-ACO* in response to CCN infection may therefore indicate that an increase in ET production is part of an infection strategy invoked by the CCN to increase susceptibility to parasitism.

In contrast to the regulation of the ET biosynthesis genes, the transcription of the gene encoding the JA receptor, COI1, displayed the opposite pattern in both interactions. In the CRKN interaction, the transcript abundance of *COI1* significantly increased at 8 dpi locally, while in the CCN interaction, the abundance of *COI1* decreased at this time point. This result supports the transcriptome study in soybean which suggests that some candidate genes in JA signalling and biosynthesis are down-regulated during well-established compatible CN infections [20]. COI1 is a SCF ubiquitin ligase that binds the JA-isoleucine conjugate and forms a complex with the jasmonate ZIM-domain (JAZ) transcriptional repressor protein. The ligase activity degrades the JAZ repressor and the MYC2 transcription factor is then free to activate a programme of jasmonate-dependent gene expression [46]. In *Arabidopsis*, RKN infection has been shown to suppress JA-dependent systemic-acquired resistance (SAR) [64], while in rice infection by *M*. *graminicola*, a systemic attenuation of both ET and JA biosynthesis is observed [59]. However, JA and ET have been shown to interact locally in a coupled manner in the infection of rice roots with *M*. *graminicola* [58]. Here, an ET-induced systemic defence pathway requires a functioning JA signalling pathway, while application of ET activates the transcription of JA biosynthesis and signalling genes. Such results have led to the postulate that RKN nematodes do not target suppression of JA signalling to induce susceptibility but rather ET and SA biosynthesis [65], and the results reported here for white clover support this. In contrast, in the interaction between the RKNs *Meloidogyne spp*., and tomato roots, JA-signalling does not play a role in defence. Instead, an intact JA signalling pathway is required for susceptibility [66]. The data from our study suggests that while there is an activation of the JA signalling pathway in response to RKN infection, there is also a marked down-regulation of ET biosynthesis so possibly compromising the plant defence response. For the CCN interaction, both the induction of ET biosynthesis and down-regulation of JA signalling occur at 8 dpi, well after the establishment of infection. Thus for the CCN interaction, the synergy between ET biosynthesis and JA signalling may become uncoupled but this occurs later in the infection time course. Thus the role of ET and JA signalling in white clover during the earlier infection stages is consistent with other CN-plant interactions. However, during the later establishment stages, the role of the hormonal cues may alter including any concomitant changes in JA biosynthesis, but this aspect of the interaction was not examined directly in this study.

## Conclusion

CRKN and CCN infection induce distinct transcriptional responses of the *Tr-KPI* gene family. The differences suggest that (i) *KPI* expression is not a key determinant of nematode resistance, and (ii) the transcriptional programme reflects differences in cellular homeostasis in response to the two distinct infection strategies. Investigation of both ET biosynthesis and JA signalling genes also reflect the differences in infection strategy and suggest that both nematode groups may uncouple the tight regulation between the two hormones.

## Supporting Information

S1 Table Sequences of primers used for qRT-PCR. (DOCX)

S2 Table GenBank accession numbers of the genes examined in this study. (DOCX)

S3 Table Mean cyst counts after 5 weeks of infection with CCN on five to eight copies of four genotypes of white clover from the breeding lines C13618 (genotypes R17 and R29) and C13704 (genotypes S23 and S27). (DOCX)
