# Supplementary material for: Transcription of Biotic Stress Associated Genes in White Clover (Trifolium repens L.) Differs in Response to Cyst and Root-Knot Nematode Infection
Source: PLoS One. 2015 Sep 22;10(9):e0137981. doi: 10.1371/journal.pone.0137981 (PMC4578895; doi:10.1371/journal.pone.0137981)
Supplement: S1 Table — (DOCX) [file pone.0137981.s001.docx]

**S1 Table**

Sequences of primers used for qRT-PCR.

| Gene | Sense | Antisense |
| --- | --- | --- |
| Tr-β-ACTIN | CGTATGAGCAAGGAGATCACTG | CATCTGCTGGAAGGTGCT |
| Tr-GAPDH | TCCAGTATTGAACGGTAAATTGAC | TCTGATTCCTCCTTGATAGCAG |
| Tr-KPI1 | GGTAACGCCATCTTCCCAG | CTACGATCTTGTAGGACAGTAACC |
| Tr-KPI2 | GGGAGATCTGGTAATGTGACAG | TCAAGGTATCAAACAACAGACTTAAT |
| Tr-KPI4 | GATATTGGAAGGCATGATGATGAG | GTCTAACAAGTGATCAGCTAACCT |
| Tr-KPI5 | CCATCCCACAAACCACCAC | GGTCCACCAATACCAACATAGC |
| Tr-COI1 | ATCGCAGCTCTAGGTTTTCC | ACAGAGACGCAGTTTCACAG |
| Tr-ACS1 | AGGTTTCGATCGAGATTTGA | CATCTGCTGGAAGGTGCT |
| Tr-ACO2 | CTTGTAAAAGGTCTCCGAGCAC | GAGGAACATCTACCCATTTACCAT |
| Tr-ACO3 | AGCATCATTCTACAACCCTGG | CAAACACAAATTTAGGATACACATTGG |
