# Supplementary material for: Transcription of Biotic Stress Associated Genes in White Clover (Trifolium repens L.) Differs in Response to Cyst and Root-Knot Nematode Infection
Source: PLoS One. 2015 Sep 22;10(9):e0137981. doi: 10.1371/journal.pone.0137981 (PMC4578895; doi:10.1371/journal.pone.0137981)
Supplement: S2 Table — (DOCX) [file pone.0137981.s002.docx]

**S2 Table**

GenBank accession numbers of genes examined in this study.

| Gene Name | GenBank Accession |
| --- | --- |
| *Tr-KPI1* | KF022195 |
| *Tr-KPI2* | KF022196 |
| *Tr-KPI4* | KF022197 |
| *Tr-KPI5* | KF022198 |
| *Tr-COI1*  *Tr-ACS1*  *Tr-ACO2*  *Tr-ACO3* | KF022210  KM881530  DQ112348  DQ112349 |
