# Supplementary material for: Transcription of Biotic Stress Associated Genes in White Clover (Trifolium repens L.) Differs in Response to Cyst and Root-Knot Nematode Infection
Source: PLoS One. 2015 Sep 22;10(9):e0137981. doi: 10.1371/journal.pone.0137981 (PMC4578895; doi:10.1371/journal.pone.0137981)
Supplement: S3 Table — (DOCX) [file pone.0137981.s003.docx]

**S3 Table**

Cyst counts after 5 weeks of infection with the clover cyst nematode on four genotypes of white clover from the breeding lines C13618 (genotypes R17 and R29) and C13704 (genotypes S23 and S27).

|  |  |  |  |  |  |
| --- | --- | --- | --- | --- | --- |
|  | **Cyst counts** | | |  |  |
|  |  |  |  |  |  |
|  | **R17** | **R29** | **S23** | **S27** |  |
|  | 0 | 55 | 56 | 87 |  |
|  | 0 | 64 | 17 | 140 |  |
|  | 6 | 235 | 59 | 139 |  |
|  | 3 | 13 | 62 |  |  |
|  | 0 | 55 | 35 | 46 |  |
|  | 0 |  | 104 | 20 |  |
|  | 0 |  | 225 |  |  |
|  | 8 | 118 | 155 |  |  |
| Mean | 3.8 | 81.3 | 81.8 | 76.5 |  |
|  |  |  |  |  |  |

R = resistant genotypes; S = susceptible genotypes
